# Faster Removal of 2-Phosphoglycolate through Photorespiration Improves Abiotic Stress Tolerance of Arabidopsis

**DOI:** 10.3390/plants8120563

**Published:** 2019-12-02

**Authors:** Stefan Timm, Franziska Woitschach, Carolin Heise, Martin Hagemann, Hermann Bauwe

**Affiliations:** 1 Plant Physiology Department, University of Rostock, Albert-Einstein-Straße 3, D-18051 Rostock, Germany; Franziska.Woitschach@med.uni-rostock.de (F.W.); Heise_Carolin@web.de (C.H.); martin.hagemann@uni-rostock.de (M.H.); hermann.bauwe@uni-rostock.de (H.B.); 2 Division of Tropical Medicine and Infectious Diseases, Center of Internal Medicine II, University Medical Center Rostock, Ernst-Heydemann-Str.6, D-18057 Rostock, Germany

**Keywords:** Arabidopsis, abiotic stress response, photosynthesis, phosphoglycolate phosphatase, photorespiration, 2-phosphoglycolate

## Abstract

Photorespiration metabolizes 2-phosphoglyolate (2-PG) to avoid inhibition of carbon assimilation and allocation. In addition to 2-PG removal, photorespiration has been shown to play a role in stress protection. Here, we studied the impact of faster 2-PG degradation through overexpression of 2-PG phosphatase (PGLP) on the abiotic stress-response of *Arabidopsis thaliana* (Arabidopsis). Two transgenic lines and the wild type were subjected to short-time high light and elevated temperature stress during gas exchange measurements. Furthermore, the same lines were exposed to long-term water shortage and elevated temperature stresses. Faster 2-PG degradation allowed maintenance of photosynthesis at combined light and temperatures stress and under water-limiting conditions. The *PGLP*-overexpressing lines also showed higher photosynthesis compared to the wild type if grown in high temperatures, which also led to increased starch accumulation and shifts in soluble sugar contents. However, only minor effects were detected on amino and organic acid levels. The wild type responded to elevated temperatures with elevated mRNA and protein levels of photorespiratory enzymes, while the transgenic lines displayed only minor changes. Collectively, these results strengthen our previous hypothesis that a faster photorespiratory metabolism improves tolerance against unfavorable environmental conditions, such as high light intensity and temperature as well as drought. In case of PGLP, the likely mechanism is alleviation of inhibitory feedback of 2-PG onto the Calvin–Benson cycle, facilitating carbon assimilation and accumulation of transitory starch.

## 1. Introduction

The characterization of a large set of photorespiratory mutants from a broad collection of phototrophs revealed photorespiration as an essential partner for oxygenic photosynthesis [1,2,3,4,5,6]. The photorespiratory pathway represents the only way to metabolize the Rubisco oxygenation reaction product 2-phosphoglycolate (2-PG; [7]) into the Calvin–Benson (CB) cycle intermediate 3-phosphoglycerate (3-PGA). Impairment of photorespiration causes 2-PG accumulation that leads to sequestration of C and P_i_, severely impeding the biosynthesis of phosphorylated intermediates and triose phosphate export from the chloroplast. 2-PG has also direct inhibitory effects on CB cycle and glycolytic enzymes [8,9,10,11]. Hence, efficient 2-PG degradation through photorespiration is particularly important for photosynthesis in the presence of O_2_. 

Despite its essential nature, the decarboxylation of glycine in the photorespiratory pathway leads to considerable losses of freshly assimilated carbon. The magnitude of these losses depends mainly on the CO_2_ and O_2_ partial pressures in the chloroplast, which can dramatically change under unfavorable environmental conditions, such as high light intensity, drought and high temperatures [12,13,14]. It is obvious that the photorespiratory flux needs to operate at higher speed in response to such conditions in order to cope with the higher 2-PG amounts. Given the CO_2_ loss during 2-PG recycling, plant research, however, aims to circumvent photorespiration in order to reduce carbon and energy losses [15,16,17]. In contrast, several reports demonstrated that an elevated flux through photorespiration could also increase photosynthesis under laboratory and field conditions [18,19,20,21] due to improved conversion of critical metabolites. 

Apart from the central role of photorespiration supporting photosynthetic CO_2_ fixation, it has been suggested that it plays also a role in the stress response of plants. For example, malfunctioning of photorespiration leads to an enhanced susceptibility of the plants against pathogen attack [22,23], and lowered tolerance towards abiotic stresses [24,25,26,27]. This is mainly because photorespiration serves as alternative energy sink under unfavorable environmental conditions regenerating ADP and NADP and, thus, decreases acceptor limitation of the light process and related ROS formation [26,28,29]. Hence, high photorespiratory flux could help to prevent the chloroplastidal electron transport chain from overreduction and, finally, photoinhibition [24]. Interestingly, NADH-dependent hydroxypyruvate reductase 1 (HPR1) protein expression significantly increases in response to water-limiting conditions [25]. This result implies that the induction of critical steps of photorespiration is a natural mechanism to enhance the metabolic flux through the pathway to dissipate excess energy under stress conditions. The efficiency of such defense mechanism might be intensified via parallel increases of the cyclic electron flow and alternative oxidase pathways [26,28,29]. Despite excess energy dissipation, it was shown intact photorespiration is required for proper stomatal regulation and as such more directly involved in stress adaptation [30,31,32]. 

Among the photorespiratory enzymes, PGLP plays a crucial role since the efficient removal of 2-PG is critical to avoid negative impacts on chloroplast function as has been demonstrated by the high oxygen sensitivity of *PGLP* knock out and knock down mutants [10]. Therefore, we aimed to gain insights if faster 2-PG removal due to *PGLP* overexpression has an impact towards the acclimation to abiotic stresses. The results obtained are discussed with respect to 2-PG toxicity on carbon utilization and allocation and the potential of faster 2-PG degradation for acclimation to abiotic environmental stresses.

## 2. Results

**Figure 1:**
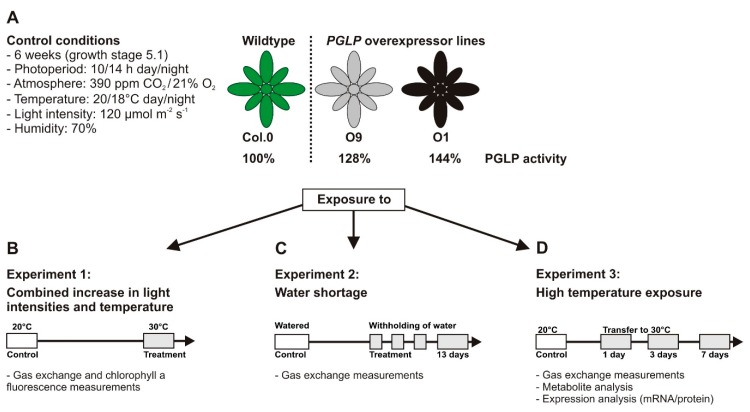
Scheme of the experimental strategy. **(A**) Following 6 weeks of growth under standard conditions, plants were used for (**B**) gas exchange and chlorophyll a fluorescence measurements. Light response curves were recorded at 20 °C (standard) and 30 °C (elevated temperature). (**C**) A subset of plants was exposed to water shortage and used for gas exchange measurements (*A*/*C_i_* curves) after 13 days or (**D**) to elevated temperature for gas exchange measurements (*A*/*C_i_* curves) after 1 and 7 days and metabolite and expression analysis.

To test whether faster 2-PG removal can improve abiotic stress tolerance, we reinvestigated the previously generated *PGLP* overexpressor lines O9 and O1. Both contain decreased absolute 2-PG amounts and display lower O_2_ inhibition of photosynthesis and altered stomatal features [10]. First, wild-type and *PGLP* overexpressor plants were grown under standard conditions (Figure 1A) and used for combined gas exchange and chlorophyll a fluorescence measurements to characterize their response to short-time light and temperature stresses (Figure 1B). Second, all genotypes were pre-grown under standard conditions and then exposed to two different long-term abiotic stresses, namely, water deficiency and higher temperatures (Figure 1C,D). 

### 2.1. PGLP Overexpressors Sustain CO_2_ Assimilation and Electron Transport at Combined High Light and Temperature

**Figure 2:**
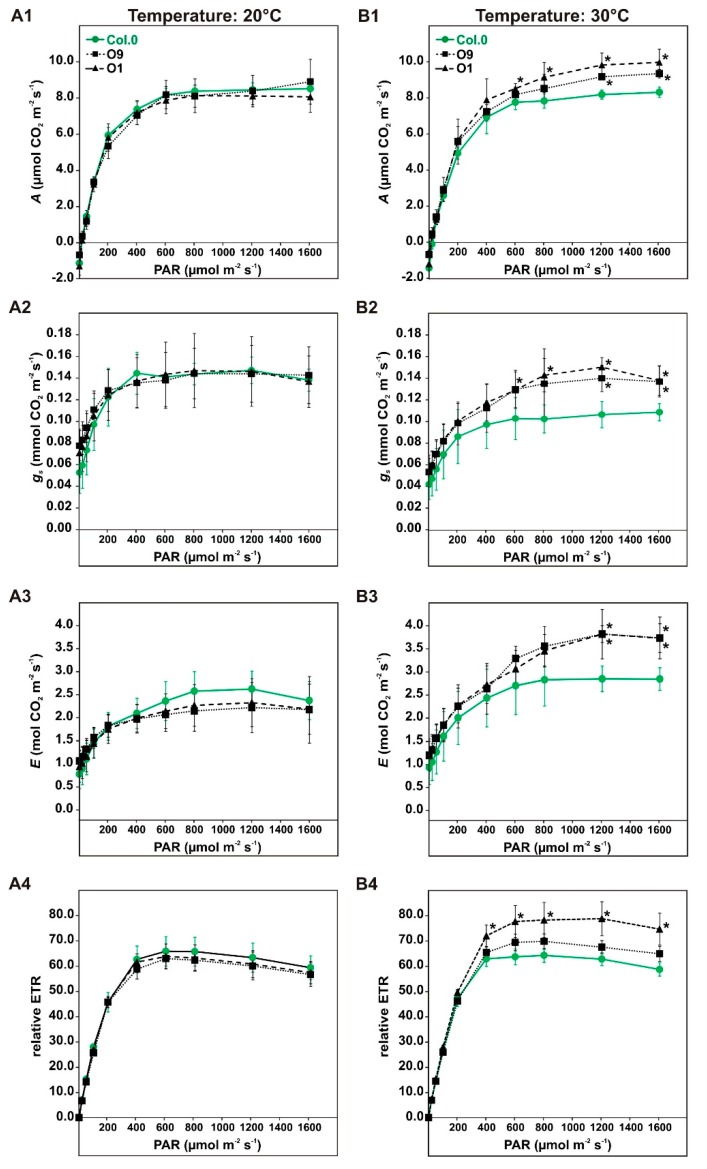
Light and temperature dependent gas exchange parameters of wild-type Arabidopsis and *PGLP* overexpressors. Plants were grown under standard conditions to growth stage 5.1 [33] and subsequently used for gas exchange measurements at (**A**) 20 °C and (**B**) 30 °C block temperature. Shown are (**A1**,**B1**) net CO_2_ uptake rates (*A*), (**A2**,**B2**) stomatal conductance (*g_s_*), (**A3**,**B3**) transpiration (*E*) and (**A4**,**B4**) relative electron transport rates (rETR). Values presented are mean values ± SD from at least four biological replicates (Col.0—green, solid line with circles, O9—black, dotted lines with squares and O1—black, dashed line with triangles). Asterisks indicate values statistically different from the wild type as determined by Student’s *t*-test (*p* < 0.05).

In a first series of experiments, we characterized the photosynthetic performance of *PGLP* overexpressors using high light intensities and elevated temperature during photosynthetic measurements. To this end, the wild type and *PGLP* overexpression lines O9 and O1 were grown under standard conditions to growth stage 5.1 [33] and then subjected to gas exchange measurements (Figure 1A,B). First, we monitored net photosynthetic CO_2_ uptake rates (*A*) in response to increasing light intensities (PAR: 0, 20, 50, 100, 200, 400, 600, 800, 1200 and 1600 µmol m^−2^ s^−1^) at the standard growth temperature of 20 °C. In parallel, stomatal conductance (*g_s_*), transpiration (*E*) and relative electron transport rates (rETR) were recorded. As shown in Figure 2A, we did not detect significant differences in any of these parameters between the transgenic lines and the wild type, which is in good agreement with previous findings [10]. However, an increase of the incubation temperature from 20 °C to 30 °C caused significant and systemic short-term changes. In comparison to the wild type, we observed significantly increased *A* at higher light intensities, ranging from 1200 to 1600 in O9 and from 600 to 1600 µmol m^−2^ s^−1^ in our best performing line O1 (Figure 2B). Similarly, both lines were able to maintain higher *g_s_* at the specified light intensities and displayed somewhat elevated *E* at 1200 and 1600 µmol^-2^ s^-1^ light (Figure 2B). Moreover, an increase in rETR was measured, but it was significant only in line O1 (400 to 1600 µmol m^−2^ s^−1^). Collectively, our results suggest that high PGLP activity is beneficial for photosynthetic CO_2_ fixation and chloroplastidal electron transport at high light intensities in conjunction with elevated temperatures. 

### 2.2. Faster 2-PG Degradation is Beneficial for Photosynthetic CO_2_ Assimilation under Water-Limiting Conditions

**Figure 3:**
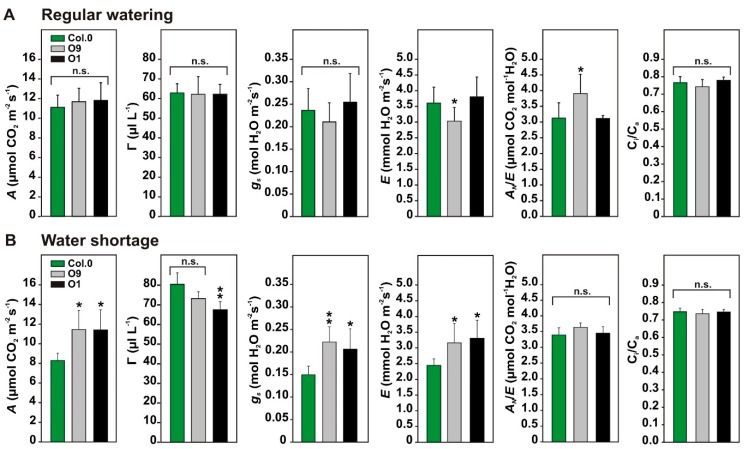
Photosynthetic parameters of wild-type Arabidopsis and *PGLP* overexpressors under water-limiting conditions. Wild-type and *PGLP* overexpressor (O9 and O1) plants were grown in one pot (28 cm diameter, 1 individual per genotype) for 6 weeks with regular watering (twice a week) under standard conditions. Following control measurements (**A**), watering was stopped, and plants measured after 13 days of withholding water (**B**). Shown are mean values ± SD from at least 5 individuals per genotype, grown as 5 technical replicates. Asterisks indicate values statistically different from the wild type as determined by Student’s *t*-test (*p* < 0.05; n. s.—not significant). Abbreviations: *A*—net CO_2_ uptake rates, *Γ*—CO_2_ compensation points, *g_s_*—stomatal conductance, *E*—transpiration, *A_N_*/*E*—water use efficiency and *C_i_*/*C_a_*—ratio of internal versus external CO_2_ concentration.

Next, we tested if the photosynthetic and stomatal features of *PGLP* overexpressors showed differences to wild-type plants under water-limiting conditions. To this end, all plants were conjointly grown under environmental controlled conditions in one pot (28 cm diameter, one plant per genotype) for 6 weeks with regular watering. Values from gas-exchange measurements at this time point served as control. Thereafter, watering was stopped, and gas-exchange measurements were repeated after 13 days under water-limiting conditions. This time point was chosen since previous studies showed upregulation of the photorespiratory pathway on the protein level 13 days after onset of water shortage [25]. As shown in Figure 3A, and in agreement with previous results [10], *PGLP* overexpressors displayed no significant change in *A*, the CO_2_ compensation points (*Γ*), *g_s_* and the ratio between the internal versus the external CO_2_ concentration (*C_i_*/*C_a_*) under control conditions. Only minor changes were observed in the transpiration rate (*E*) and the water use efficiency (*A_N_*/*E*) in line O9. After 13 days of withholding water, wild-type plants compromised photosynthetic performance, whereas both overexpressor lines displayed significantly higher *A*, *g_s_* and *E* levels, while *Γ* was decreased (significant in line O1). The *C_i_*/*C_a_* and *A_N_*/*E* parameters remained almost unchanged (Figure 3B). In summary, overexpression of *PGLP* permits a longer maintenance of the photosynthetic capacity under water-limiting conditions. 

### 2.3. High PGLP Activity is Beneficial for Photosynthesis at Increased Growth Temperatures

**Figure 4:**
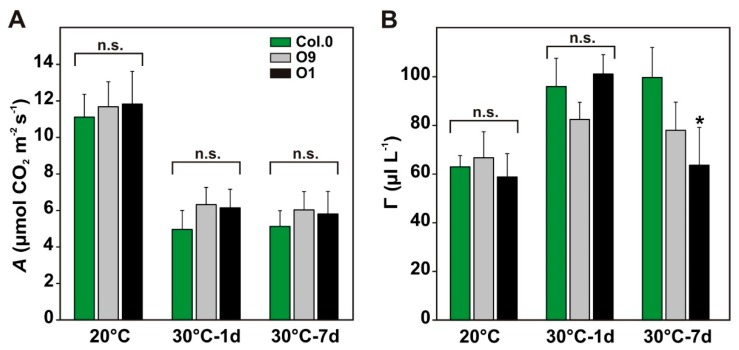
Photosynthetic parameters of wild-type Arabidopsis and *PGLP* overexpressors exposed to 30 °C. Wild-type and *PGLP* overexpressor (O9 and O1) plants were grown under standard conditions (20 °C) for 6 weeks following exposure to elevated temperature (30 °C). Gas exchange measurements were carried out to determine (**A**) CO_2_ assimilation rates (*A*) and (**B**) net CO_2_ compensation points (*Γ*) under control conditions (20 °C) and after 1 and 7 days after the transfer to 30 °C. Shown are mean values ± SD from at least 4 biological replicates per genotype. Asterisks indicate values statistically different from the wild type as determined by Student’s *t*-test (*p* < 0.05; n. s.—not significant).

Next, we aimed to know whether improved maintenance of photosynthesis was restricted to water-limiting conditions (Figure 3B) or if it also occurs in response to other abiotic stresses in *PGLP* overexpressor lines. Therefore, we tested increased growth temperature, which is anticipated to stimulate 2-PG production. To this end, the transgenic lines and the wild type were grown for 6 weeks under standard conditions and then exposed to elevated temperatures (30 °C; Figure 1D). As proxies for their capability to acclimate to higher temperatures, we characterized photosynthetic parameters such as *A* and *Γ* under control conditions and after one and 7 days at 30 °C. As before, we did not measure significant differences between the genotypes under control conditions. As expected, transfer of the plants to 30 °C decreased *A* and increased *Γ*. *A* decreased to the same extends in the transgenic lines and the wild type (Figure 4A). However, after 7 days exposure to 30 °C, *Γ* is significantly decreased in both transgenic lines, reaching almost control levels, while it remained at a similarly high level in wild type as found after only one day at 30 °C (Figure 4B, significant in O1). This change in line O1 was accompanied by significantly increased *g_s_* (Col.0—0.1032 ± 0.0202 versus O1—0.1985 ± 0.0726*) and *E* (Col.0—1.67 ± 0.28 versus O1—2.96 ± 0.86*), which remained similar between O9 and the wild type (*g_s_*—0.1266 ± 0.0250; *E*—1.92 ± 0.33). Collectively, O1, the line with lowest 2-PG contents [10], has a significant advantage over the wild type after exposure to elevated growth temperatures for one week. This beneficial effect is likely not due to enhanced CO_2_ fixation, but rather due to improved carbon utilization as suggested by the lower *Γ*. 

### 2.4. Improved 2-PG Degradation Translates to Higher Transitory Starch Stocks under Temperature Stress 

**Figure 5:**
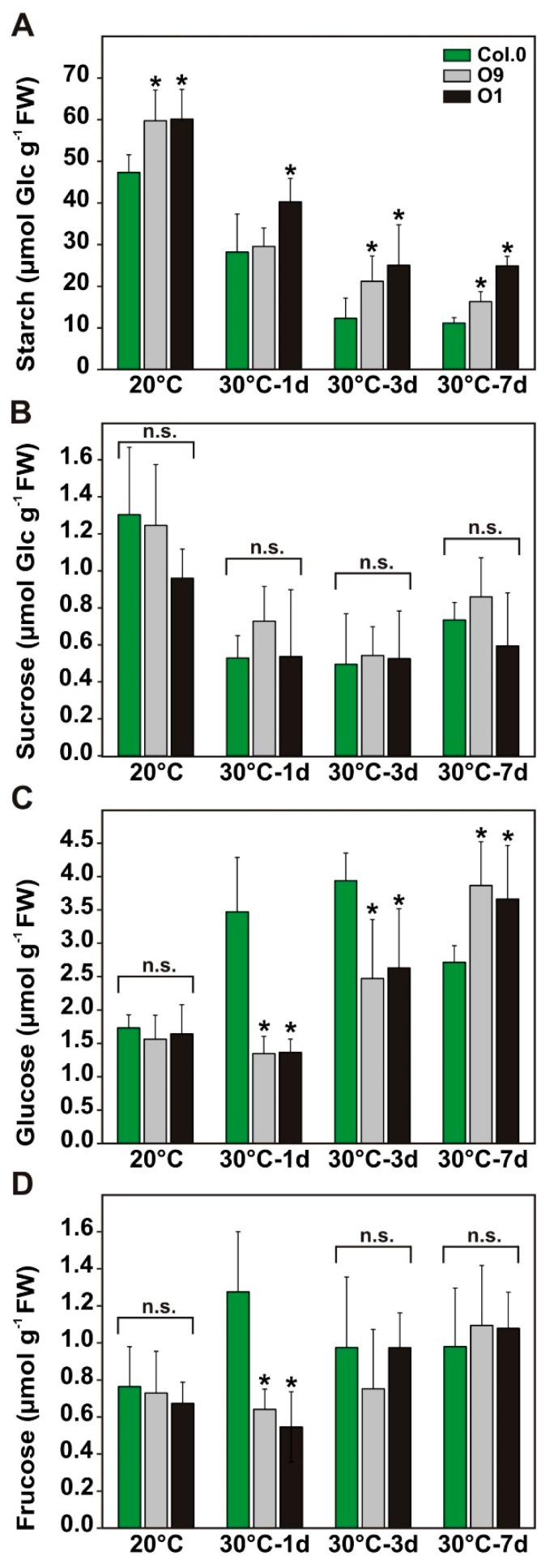
Carbohydrate contents of wild-type Arabidopsis and *PGLP* overexpressors exposed to 30 °C. Wild-type and *PGLP* overexpressor (O9 and O1) plants were grown in standard conditions (20 °C) for 6 weeks following exposure to elevated temperature (30 °C). Leaf material was harvested at the end of the day (9 h illumination) to determine absolute (**A**) starch, (**B**) sucrose, (**C**) glucose and (**D**) fructose contents under control conditions and after 1, 3 and 7 days after the transfer to 30 °C using gas chromatography. Shown are mean values ± SD from at least 4 biological replicates per genotype. Asterisks indicate values statistically different from the wild type as determined by Student’s *t*-test (*p* < 0.05; n. s.—not significant).

Given that 2-PG levels are inversely correlated with starch accumulation [10], we quantified the amounts of starch and soluble sugars at the end of the day (EoD) in leaf-material from the temperature shift experiment. In agreement with our previous report [10], the overexpressors O9 and O1 contained significantly elevated starch contents (~27%) at EoD under control conditions compared to the wild type (Figure 5A). After the transfer to 30 °C, wild-type starch levels significantly drop compared to the control, which is in agreement with other reports [34]. Notably, both transgenic lines were able to maintain higher starch accumulation after the temperature increase, which was clearly pronounced 7 days after the shift to 30 °C. At this time point, O9 starch increased to about 46% and O1 to about 123% (Figure 5A). About the contents of soluble sugars, we did not find significant change in sucrose (Figure 5B). Glucose was similar in all plants at 20 °C. However, it significantly decreases in both lines after one to three days at 30 °C and increased again after 7 days at 30 °C (Figure 5C). Changes in fructose were only seen after one day at 30 °C, when both overexpressor lines displayed a significant drop compared to wild type (Figure 5D). Collectively, our results suggest that faster 2-PG removal and sustained photosynthesis are beneficial for carbon allocation towards transitory starch under control as well as stress conditions, without impacting steady-state sucrose amounts.

### 2.5. Minor Changes in the Amino and Organic Acid Contents during Temperature Stress

**Figure 6:**
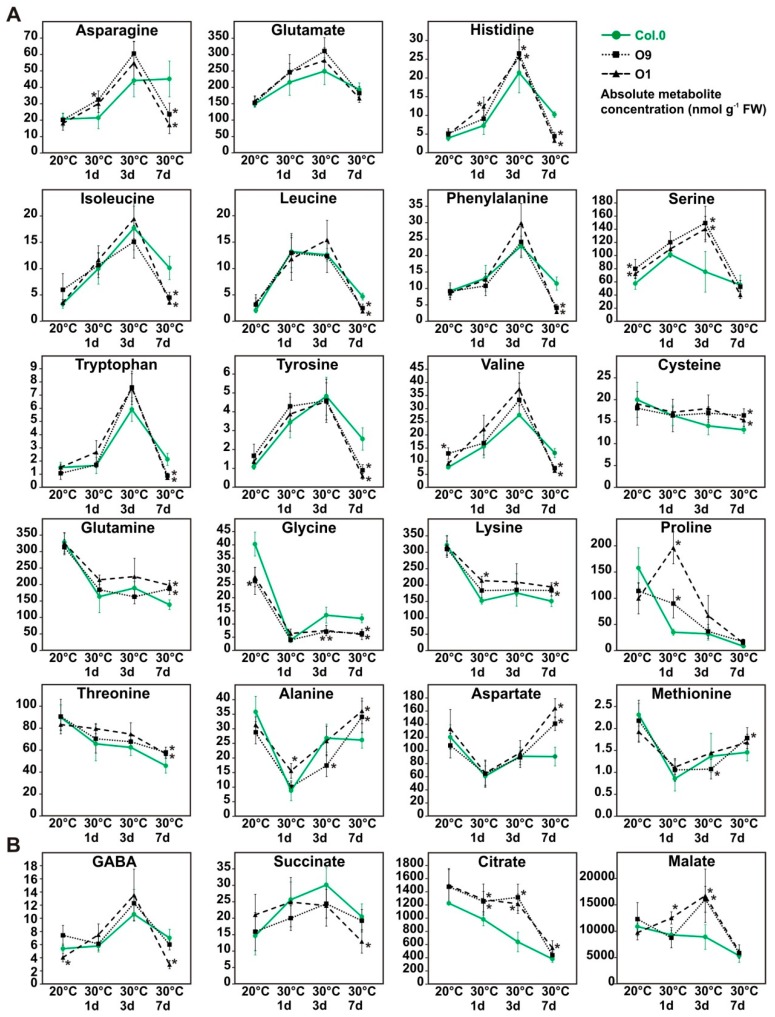
Amino acids and organic acid of wild-type Arabidopsis and *PGLP* overexpressors exposed to 30 °C. Wild-type and *PGLP* overexpressor (O9 and O1) plants were grown in under standard conditions (20 °C) for 6 weeks following exposure to elevated temperature (30 °C). Leaf material was harvested at the end of the day (9 h illumination) to determine absolute (**A**) amino acid and (**B**) organic acids contents under control conditions and after 1, 3 and 7 days after the transfer to 30 °C via liquid chromatography coupled to tandem mass spectrometry (LC-MS/MS). Shown are mean values ± SD of absolute metabolite concentrations (nmol g^−1^ FW) from at least 4 biological replicates per genotype (Col.0—green, solid line with circles, O9—black, dotted lines with squares and O1—black, dashed line with triangles). Asterisks indicate values statistically different from the respective wild-type time point as determined by Student’s *t*-test (*p* < 0.05).

Considering the observed changes among carbohydrates, we were interested to which extent *PGLP* overexpression could also affect amino and organic acid levels in response to increased growth temperature. To this end, we used the same leaf-material harvested at EoD (9 h illumination, 20 °C and after 1, 3 and 7 days in 30 °C) for metabolite analysis by liquid chromatography coupled to tandem mass spectrometry (LC-MS/MS). Among the amino acid profiles (Figure 6A), we found three general accumulation patterns. Ten amino acids (group A: asparagine, glutamate, histidine, isoleucine, leucine, phenylalanine, serine, tryptophan, tyrosine and valine) increased in their abundance after the transfer to elevated temperature, with highest amounts after three days at 30 °C, but distinctly drop at day 7 (Figure 6A) to almost control levels. Six amino acids (group B: cysteine, glutamine, glycine, lysine, proline and threonine) were characterized by a gradual decrease over the course of the entire experiment. A third pattern, comprising three amino acids (group C: alanine, aspartate and methionine), showed an initial drop at day one at 30 °C and then a subsequent increase until the initial levels at 20 °C was reached (Figure 6A). In general, the observed pattern was similar in wild-type and overexpressor plants. However, glycine, an amino acid directly related to photorespiration, was found in lower amounts under control conditions and after three and 7 days at 30 °C in both overexpressor lines (Figure 6A). In addition, many amino acids showed changed levels at day 7 at 30 °C. For example, almost all of group A amino acids were decreased in the overexpressors after 7 days of temperature stress (Figure 6A), whereas group B and C representatives displayed slight increases after 7 days. Proline, as an exception, was higher in both lines one day after the shift to elevated temperature. Concerning the organic acids, we found that citrate and malate followed the same accumulation kinetics as group B amino acids, at least in the wild type (Figure 6B). Both tend to decrease over time but were significantly increased in the transgenic lines one (except O9 malate) and three days after the shift to 30 °C (Figure 6B). Compared to that, GABA and succinate fluctuate like group A amino acids, without major change in the different genotypes.

### 2.6. Expression of Photorespiratory Proteins Increases after High Temperature Exposure in Wild Type but not in PGLP Overexpressor Lines

**Figure 7:**
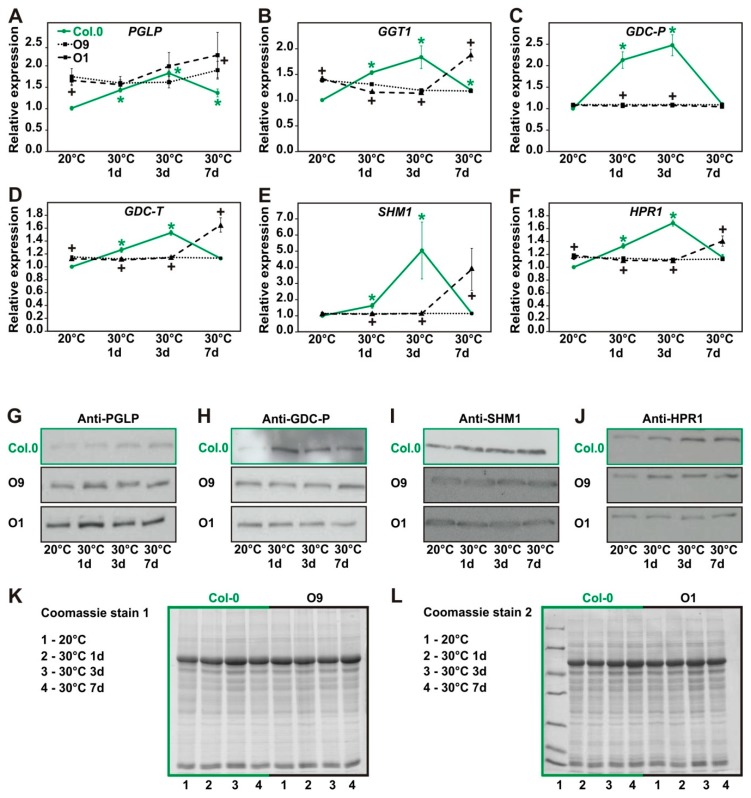
Expression of selected photorespiratory gene on the mRNA and protein level in wild-type Arabidopsis and *PGLP* overexpressors exposed to 30 °C. Wild-type and *PGLP* overexpressor (O9 and O1) plants were grown in under standard conditions (20 °C) for 6 weeks following exposure to elevated temperature (30 °C). Leaf material was harvested at the end of the day (9 h illumination) to analyse mRNA and protein expression of selected photorespiratory genes under control conditions (20 °C) and after 1, 3 and 7 days after the transfer to 30 °C. Shown are: mRNA expression of (**A**)—*PGLP*, (**B**)—*GGT1*, (**C**)—*GDC-P*, (**D**)—*GDC-T*, (**E**)—*SHM1*, and (**F**)—*HPR1* and protein amounts of (**G**)—PGLP, (**H**)—GDC-P, (**I**)—SHM1 and (**J**)—HPR1. Coomassie stains (10 µg protein per lane) are shown in (**K**) and (**L**) as loading controls. Values are means ± SD from at least 3 biological replicates per genotype (Col.0—green, solid line with circles, O9—black, dotted lines with squares and O1—black, dashed line with triangles). Asterisks indicate values statistically different from the wild type control at 20 °C and plusses of the transgenic lines to the respective wild-type time point as determined by Student’s *t*-test (*p* < 0.05).

Previously, it was reported that the expression and amounts of some photorespiratory genes and proteins, respectively, changed in response to abiotic stresses [25,35]. Therefore, we analyzed the expression of selected photorespiratory genes and proteins during the high temperature treatment using quantitative real-time polymerase chain reaction (qRT-PCR) and immunoblotting, respectively. As shown in Figure 7, mRNA levels of several photorespiratory genes (*PGLP1*, glutamate:glyoxylate aminotransferase 1 (*GGT1*), glycine decarboxylase P and T protein (*GDC-P* and *GDC-T*), serine hydroxymethyltransferase 1 (*SHM1*) and peroxisomal hydroxypyruvate reductase 1 (*HPR1*)) were significantly elevated in wild-type leaves after one and three days at 30 °C, while after 7 days their expression levels returned almost to the initial levels at 20 °C (Figure 7). To prove, whether these transcriptional alterations translated also to changes in the protein abundances, we exemplarily analyzed PGLP1, GDC-P, SHM1 and HPR1 protein amounts at the same time points. As observed before for mRNA expression, all four proteins increased in wild type after exposure to 30° (Figure 7). In contrast, both *PGLP* overexpression lines did not display the increased expression of photorespiratory genes and proteins. As expected, lines O9 and O1 showed significantly increased *PGLP* expression on the mRNA and protein level already under control conditions and only minor alterations in course of the temperature treatment (Figure 7A). However, all the other photorespiratory genes analyzed showed constantly lower mRNA and only very minor changes in the protein levels during the entire experiment. Only the genes *GGT1*, *GDC-T*, *GDC-P* and *HPR1* showed higher mRNA amounts in line O1 after 7 days at 30 °C (Figure 7B,D,E,F). From these results we conclude that *PGLP* overexpression somehow prepares the plants to cope with the temperature stress without a coordinative upregulation of other genes and enzymes involved in photorespiration. 

## 3. Discussion

The main function of the photorespiratory pathway is the efficient removal of critical intermediates, especially 2-PG, which severely inhibits carbon fixation and allocation [6,10]. Furthermore, it has been suggested that photorespiration plays a role in the abiotic stress response of plants [25,26,27]. Therefore, we hypothesized that an increased photorespiratory flux might facilitate plant abiotic stress tolerance. To test this hypothesis, we used transgenic lines overexpressing photorespiratory *PGLP*, previously shown to fix more carbon at high photorespiratory pressures and to display improved starch metabolism and stomatal movements [10]. 

Consistent with our hypothesis, we found improved photosynthetic performance after short-term and long-term abiotic stresses. In a first series of experiments, *PGLP* overexpression lines displayed higher CO_2_ assimilation accompanied with improvements in other gas exchange parameters after increasing the temperature to 30 °C during short-term high light treatment, anticipated to promote 2-PG production (Figure 2B). The maintenance of higher photosynthesis in *PGLP* overexpressors under short-term temperature stress is likely through diminished inhibition of the CB cycle by 2-PG, but we cannot rule out that increased photorespiration eventually helps to prevent the chloroplastidal electron transport chain from overreduction as suggested before [26,28,29]. Similar results were obtained if photosynthesis of wild-type and *PGLP* overexpressor plants was characterized under water-limiting conditions. Water shortage promotes stomatal closure [36] and eventually increases 2-PG levels due to a higher RuBP fraction being oxidized. Notably, both transgenic lines showed significant improvements in photosynthetic parameters after 13 days of water shortage (Figure 3). Interestingly, we did not only measure higher CO_2_ assimilation and lower CO_2_ compensation points, but also higher *g_s_*, indicating altered stomatal movements (Figure 3B). Given that photorespiration is also involved in proper guard cell metabolism [30,31], an optimized flux through the pathway in mesophyll cells could be beneficial for these specialized cells, too. Accordingly, it is likely to assume that the altered leaf-carbohydrate metabolism, in particular starch biosynthesis, can facilitate allocation of carbon from the mesophyll to the guard cells in order to enhance their energy supply. It should be noted that *PGLP* overexpression is driven through the *ST-LSI* promoter, therefore, changes in *PGLP* expression are not restricted to the mesophyll cells. Hence, changes in *g_s_* could also be directly caused by altered PGLP activity in guard cells. 

Finally, long-term exposure at elevated temperatures was analyzed in more detail. This was done for three reasons: (i) elevated temperatures favor 2-PG production, (ii) higher PGLP activities were found to be beneficial for photosynthesis on a short-term (Figure 2), and (iii) future climate change scenarios predict an increase in temperature on a global scale [13,14]. Consequently, higher *PGLP* activity could eventually be a positive trait for plant engineering. In agreement with the short-term exposure to high light and elevated temperature (Figure 2) and growth under water-limiting conditions (Figure B), the overexpressor line O1 maintained higher photosynthesis after long-term exposure to elevated temperature (Figure 4). This change was accompanied by higher *g_s_* and increased transpiration, again suggesting changes in stomatal movements. Given the strong impact 2-PG has on CO_2_ fixation and carbohydrate utilization and allocation [6,10], we quantified amounts of starch and the soluble sugars sucrose, glucose and fructose (Figure 5). In line with previous findings [10], overexpressors of *PGLP* store somewhat more starch under standard conditions, without a significant impact on sucrose synthesis. Interestingly, these trends were kept after the shift to elevated temperature until day 7 at 30 °C. These data indicate faster 2-PG removal facilitates carbon allocation to starch biosynthesis also under the stress conditions. However, we cannot neglect the possibility that starch is degraded somewhat less fast in the transgenic lines. The assumption of faster starch synthesis is supported by the decreased amounts of soluble sugars in both lines during the first days at 30 °C. However, after 7 days at 30 °C, this trend became reversed, which might be a compensatory reaction of carbon metabolism under long-term temperature stress. The effects of *PGLP* overexpression seem to be mainly restricted to alterations in photosynthesis and carbohydrate metabolism, since amino and organic acid contents showed only minor changes.

Acclimation to elevated temperatures obviously activates the photorespiratory activity in wild-type plants, because a coordinated increase of mRNAs and proteins for many photorespiratory enzymes was observed (Figure 7), which is consistent with previous reports [25]. Given the lack of response on the expression of the photorespiratory enzymes in the transgenic lines, one might speculate upregulation of *PGLP*, and in turn lowering the steady-state content of 2-PG, already acts as signal to indicate sufficient acclimation of the photorespiratory flux. Additionally, this result once more demonstrates the importance of fast 2-PG removal via photorespiration, which might be sufficient to cope with the temperature stress. Furthermore, 2-PG could also play a regulatory role in the stress acclimation as shown for serine before [37]. Direct involvement of 2-PG as inducer for the transcription of genes involved in CO_2_ uptake was observed in cyanobacteria [38], but has not yet reported for plants. However, the *PGLP* knock out mutant showed strong and specific alterations in gene expression after a shift from high to low CO_2_ [31]. Therefore, it cannot be excluded completely that 2-PG mediated transcriptional reprogramming mechanisms exist in plants. 

## 4. Material and Methods

### 4.1. Plant Material and Standard Growth Conditions

The generation of stable T4-generations of *Arabidopsis thaliana* (Arabidopsis) lines (ecotype Columbia 0, Col-0) overexpressing the photorespiratory phosphoglycolate phosphatase 1 (*PGLP1*, At5g36700, EC 3.1.3.18) was described previously [10]. Lines with an approximately 28% (O9) and 44% (O1) increase in PGLP activity compared to the wild type were used during this study. Prior plant cultivation, seeds of all genotypes were surface sterilized with chloric acid, sown on a soil (Type Mini Tray; Einheitserdewerk, Uetersen, Germany) and vermiculite mixture (4:1) and incubated at 4 °C for at least two days to break dormancy. Subsequently, plants were grown under environmental controlled conditions in growth cabinets (SANYO, Osaka, Japan; CLF Plant Climatics, Wertingen, Germany) with the following conditions as a standard: photoperiod - 10/14 h day/night-cycle, temperature - 20/20 °C day/night-cycle, photon flux density of ~120 µmol m^−2^ s^−1^, 70% relative humidity, 0.039% CO_2_ in air (Figure 1A). During growth, plants were regularly watered with 0.2% Wuxal liquid fertilizer (Aglukon, Düsseldorf, Germany). 

### 4.2. Stress Conditions

Wild-type and *PGLP* overexpression plants were grown under standard conditions (Figure 1A), following exposure to two different stress conditions. First, simulating water-limiting conditions, all genotypes were grown conjointly in one pot (28 cm in diameter, 1 plant per genotype, 5 technical replicates) for 6 weeks with regular water supply to allow for a high level of comparability. After control experiments were carried out, watering was stopped, and experiments performed at intervals specified in the manuscript text. Second, simulating temperature stress, all genotypes were grown under standard conditions for 6 weeks and control experiments carried out. Subsequently, all plants were exposed to elevated temperatures (30 °C) with otherwise equal conditions. Photosynthetic measurements were performed, and leaf-material harvested after 1, 3 and 7 days in 30 °C.

### 4.3. qRT-PCR Analysis and Immunological Studies

To follow the expression of selected photorespiratory genes and proteins we harvested leaf-material at the end of the day (9 h illumination) during temperature transition under control conditions (20 °C) and after 1, 3 and 7 days in 30 °C. For gene expression analysis total leaf RNA was extracted from ~100 mg tissue (pooled from three biological individuals) and ~2.5 µg used to synthesize cDNA (Nucleospin RNA plant kit, Macherey-Nagel; RevertAid cDNA synthesis kit, MBI Fermentas). Prior to qRT-PCR analysis, cDNA amounts were calibrated by RT-PCR according to signals from 432-bp fragments of the constitutively expressed 40S ribosomal protein *S16* gene, with oligonucleotides P444 [5′-GGC GAC ACA ACC AGC TAC TGA-3′] and P445 [5′-CGG TAA CTC TTC TGG TAA CGA-3′]. Detection and normalization of gene expression were performed as described previously (Timm et al., 2013), and mRNA amounts of *PGLP1* (P393 [5′-CAG AAT GGC GGT TGT AAG AC-3′] and P394 [5′-GGC TCC CTA ATT TGC TAT GC-3′]; 328 bp), *GGT1* (P405 [5′-CGT TGC TCA GGC TCG TTC TC-3′] and P406 [5′-CCA CCT CGC TGT CCA CAT TC-3′]; 336 bp), *GDC-P1* (P366 [5′-AGC AAA TCC GTA GCC ATC AC-3′] and P413 [5′-TAT GTC CAA TGC GTC GCT TC-3′]; 327 bp), *GDC-T* (P367 [5′-GCA ATC AAT AAC CCG TCG TC-3′] and P368 [5′-TCA ATG GCA CCT CCT TTC TC-3′]; 363 bp), *SHM1* (P395 [5′-GCC CAG TGA AGC TGT TGA TG-3′] and P396 [5′-AGT TGG CAG GAG ATC CAG AC-3′]; 365 bp) and *HPR1* (P397 [5′-GGC TGA ACT AGC TGC TTC TC-3′] and P398 [5′-GCA CCG GGT GAA GAC TTA TC-3′]; 360 bp) quantified accordingly using the oligonucleotide combinations given in brackets after each gene. Abundances of selected photorespiratory proteins were analyzed by immunoblotting. Briefly, total leaf proteins were extracted from ~100 mg leaf tissue (pooled from three biological individuals) and 10 µg separated by SDS-PAGE followed by immunoblotting according to standard protocols. Alterations in protein expression were visualized using specific antibodies against PGLP1, GDC-P, SHM1 and HPR1 [38].

### 4.4. Determination of Starch and Metabolite Analysis

Starch contents were measured enzymatically as described previously [39] from ~50 mg of leaf tissue harvested at EoD (9 h illumination) from at least four biological replicates per genotype. The soluble fraction of the extraction procedure was further subjected to gas chromatography (GC) analysis to quantify sucrose, glucose and fructose as described previously [40]. Amino acids and organic acids were essentially quantified on a high-performance liquid chromatograph mass spectrometer LCMS-8050 system (Shimadzu, Japan) as described recently [41] from 50 mg leaf-tissue harvested at the end of the day (9 h of illumination). The compounds were identified and quantified using the multiple reaction monitoring (MRM) values given in the LC-MS/MS method package and the LabSolutions software package (Shimadzu, Japan). Authentic standard substances (Merck, Germany) at varying concentrations were used for calibration and peak areas normalized to signals of the internal standard (2-(N-morpholino)-ethanesulfonic acid - MES). 

### 4.5. Gas Exchange and Chlorophyll a Fluorescence Measurements 

All gas exchange parameters were determined in a 6-h time period between 2 h after onset and 2 h prior offset of illumination on a Li-Cor-6400 gas exchange system (LI-COR, Lincoln, NE, USA) using fully expanded leaved from plants at growth stage 5.1 [33]. Prior the actual measurement, leaves were pre-adapted to the measuring chamber for at least 10 min. To determine net CO_2_ compensation points (*Γ*), *A*/*C_i_* curves (400, 300, 200, 100, 50, 20, 0, 400 ppm CO_2_) were recorded with the following conditions: photon flux density = 1000 µmol m^−2^ s^−1^, chamber temperature = 20 °C, flow rate = 300 µmol s^−1^ and relative humidity = 60% to 70%. Fluorescence light response curves (PAR: 1600, 1200, 800, 600, 400, 200, 100, 50, 20 and 0 µmol m^−2^ s^−1^) were measured at two different block temperatures (20 °C and 30 °C) with the following conditions: CO_2_ concentration = 400 ppm; flow rate = 300 µmol s^−1^ and relative humidity = 60 to 70%. Relative rates of electron transport around PSII at a given light intensity (PAR) were assessed by the formula ETR = Y_PSII_ × PAR × 0.84 × 0.5. The factors are based on the assumptions that 84% of the incident quanta are absorbed by the leaf (factor 0.84) and that the transport of one electron by the two photosystems requires the absorption of two quanta (factor 0.5). Y_PSII_ (effective quantum yield of photosystem II) was calculated as described previously [42] using the following formula:

### 4.6. Statistical Analysis

If values were described to be significantly different from the control within the text, the differences have been determined due to the performance of the two tailed Student′s *t*-test algorithm incorporated into Microsoft Excel 10.0 (Microsoft, Seattle, WA, USA).

### 4.7. Accession Numbers

The Arabidopsis Genome Initiative database contains sequence data from this article under the following accession numbers: *PGLP1* (At5g36700), *GDC-P1* (At4g33010), *GDC-T* (At1g11680), *SHM1* (At4g37930), *HPR1* (At1g68010), *GGT1* (AT1G23310), and 40S ribosomal protein *S16* (At2g09990).

## 5. Conclusions

In summary, we provided evidence that upregulation of PGLP activity leading to lowered 2-PG contents, is beneficial for maintaining photosynthesis under abiotic stresses. This is likely due to the removal of 2-PG-mediated negative metabolic feedback on central enzymes of the CB cycle and carbon export from the chloroplast. We suggest that an optimized photorespiratory flux can thus stabilize the production and allocation of organic carbon under unfavorable environmental conditions. The strategy presented here might have further implications for plant engineering approaches to generate highly productive and more stress-resistant plants for future climate scenarios.
